# Sunflower Resistance to Broomrape (*Orobanche cumana*) Is Controlled by Specific QTLs for Different Parasitism Stages

**DOI:** 10.3389/fpls.2016.00590

**Published:** 2016-05-10

**Authors:** Johann Louarn, Marie-Claude Boniface, Nicolas Pouilly, Leonardo Velasco, Begoña Pérez-Vich, Patrick Vincourt, Stéphane Muños

**Affiliations:** ^1^LIPM, Université de Toulouse, INRA, CNRS, Castanet-TolosanFrance; ^2^Instituto de Agricultura Sostenible-Consejo Superior de Investigaciones Cientifícas, CordobaSpain

**Keywords:** broomrape, sunflower, resistance, QTL mapping, *Orobanche cumana*, plant–plant interaction, candidate genes, parasitic weeds

## Abstract

*Orobanche cumana* (sunflower broomrape) is an obligatory and non-photosynthetic root parasitic plant that specifically infects the sunflower. It is located in Europe and in Asia, where it can cause yield losses of over 80%. More aggressive races have evolved, mainly around the Black Sea, and broomrape can rapidly spread to new areas. Breeding for resistance seems to be the most efficient and sustainable approach to control broomrape infestation. In our study, we used a population of 101 recombinant inbred lines (RILs), derived from a cross between the two lines HA89 and LR1 (a line derived from an interspecific cross with *Helianthus debilis*). Rhizotrons, pots and field experiments were used to characterize all RILs for their resistance to *O. cumana* race F parasitism at three post vascular connection life stages: (i) early attachment of the parasite to the sunflower roots, (ii) young tubercle and (iii) shoot emergence. In addition, RIL resistance to race G at young tubercle development stage was evaluated in pots. The entire population was genotyped, and QTLs were mapped. Different QTLs were identified for each race (F from Spain and G from Turkey) and for the three stages of broomrape development. The results indicate that there are several quantitative resistance mechanisms controlling the infection by *O. cumana* that can be used in sunflower breeding.

## Introduction

The parasitic weed *Orobanche cumana* (sunflower broomrape) is an obligatory and non-photosynthetic root parasitic plant of the sunflower (*Helianthus annuus* L.) and is a substantial threat in Europe, especially in countries around the Black Sea and in Spain (Molinero-Ruiz et al., 2013). *O. cumana* has a negative effect on sunflower development. The infected plants are smaller, the sunflower head diameter is reduced and up to 80% of yield losses are observed (Alcántara et al., 2006; Duca, 2015).

Unlike other weedy *Orobanche* species, for which genetic resistance in the host is of quantitative nature (horizontal), genetic resistance to *O. cumana* in the sunflower is in most cases qualitative or vertical (Fernández-Martínez et al., 2015). For this reason, *O. cumana* populations are commonly classified into physiological races (Vranceanu et al., 1980) that periodically surpass all the available resistance sources. Eight races of *O. cumana*, A through H, have been reported thus far, with races F, G, and H commonly reported in several countries (Kaya, 2014). Different mechanisms have been described that might determine the rapid emergence of new races of *O. cumana* including recombination and increase of genetic diversity, mutation and selection within specific gene pools, or gene flow between wild and weedy *O. cumana* populations (Pineda-Martos et al., 2013, 2014).

Several methods of broomrape control are available with more or less efficiency. Different crop management solutions can be used: soil solarization (Mauromicale et al., 2005), biological control (Thomas et al., 1998; Louarn et al., 2012) and the use of herbicides such as imidazolinone combined with herbicide-tolerant sunflower hybrids (Tan et al., 2005). However, breeding for genetic resistance remains the most efficient method. The first introgression of genetic resistance to broomrape on the sunflower was conducted in the former USSR (Pustovoit, 1966). Genetic resistance has been characterized in wild *Helianthus* spp., and introgression of the resistance gene from interspecific crosses has been reported (Jan and Fernandez-Martínez, 2002; Velasco et al., 2007). Even if neither quantitative trait loci (QTLs) nor major genes were mapped for the resistance to *O. cumana* race G, Velasco et al. (2012) showed that the resistance (from *H. debilis* subsp. *tardiflorus*) to the race G of *O. cumana* was dominant and controlled by a single locus in their population. Several major *Or* resistance genes controlling the resistance to specific *O. cumana* races have been used in breeding programs (Fernández-Martínez et al., 2008). However, there are only two reports for the molecular genetic mapping of resistance loci. The first one concerns the *Or5* gene conferring resistance to race E (Lu et al., 2000; Tang et al., 2003; Pérez-Vich et al., 2004). The second report details the mapping of QTLs for resistance to *O. cumana* race F (Pérez-Vich et al., 2004). Six QTLs controlling the number of *O. cumana* emergences in the field have been detected on five linkage groups (LG). No genes have been cloned, and the molecular mechanisms involved in the resistance mechanisms remain unknown.

**FIGURE 1:**
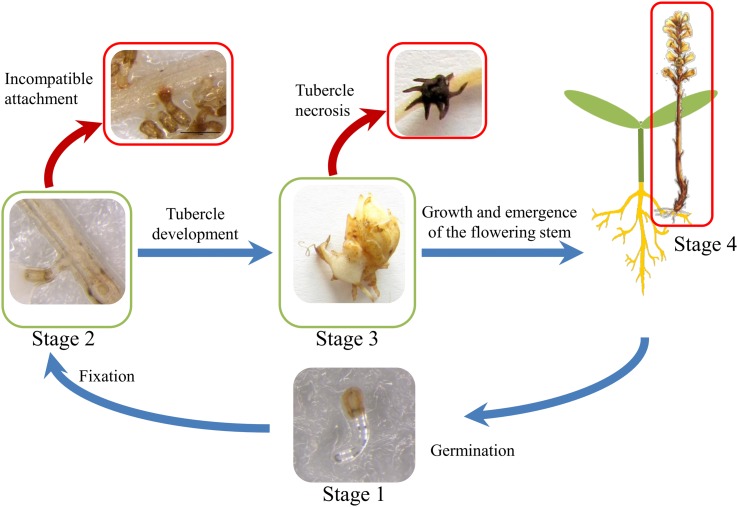
**Life cycle of *O. cumana*.** Stage 1 corresponds to broomrape seed germination induced by sunflower root exudates. During stage 2, *O. cumana* radicle attaches, invades and connects the vascular system of the sunflower root by means of haustorium. Once *O. cumana* is attached and the nutrient diversion towards the parasite is established, *O. cumana* develops a tubercle (stage 3). During the stage 4, the stem emerges through the soil surface with the subsequent onset of flowering. The red arrows show putative resistance mechanism to *O. cumana* at stage 2 (incompatible attachment) and at stage 3 (tubercle necrosis).

The life cycle of broomrape is composed of several steps from seed germination to plant flowering and seed production (Gibot-Leclerc et al., 2012; Yang et al., 2015). These steps can be roughly classified into four stages (**Figure 1**). During stage 1, the germination of the *O. cumana* seeds is induced by the host. Germination is one of the most studied steps of the broomrape life cycle. The molecules secreted by the host root system play a major role in the induction of broomrape germination (Fernández-Aparicio et al., 2009a). Two main types of molecules exuded by sunflower roots are known to induce *O. cumana* seeds germination: strigolactones and sesquiterpene lactones (Cook et al., 1966; Joel et al., 2011; Yoneyama et al., 2011; Raupp and Spring, 2013). Germination is followed by stage 2, in which the fixation of the parasite to the sunflower root, root penetration and establishment of vascular connections between the parasite and the host is achieved by means of the haustorium developed at the tip of *O. cumana* radicle (Hassan et al., 2004). After vascular connection, broomrape begins to derive phloemic flow acting as a strong nutrient sink. During stage 3, nutrient storage organ called tubercle develop quickly at the attachment point from which an underground shoot will eventually develop (Aly et al., 2009). The last stage is the above ground stage 4, which begins with the emergence of *O. cumana* from the ground and ends with flowering and seed production and dispersal. During the broomrape life cycle, several resistance mechanisms operating at the pre-attachment, pre-haustorial, or post-haustorial stages of the parasite-host interaction have been reported (Fernández-Martínez et al., 2015). These include mechanisms acting at the pre-attached stage such as decreased strigolactone exudation by host roots (Jamil et al., 2011; Fernández-Aparicio et al., 2014), or mechanisms such as cell wall deposition, vessel occlusion, broomrape cellular disorganization occurring during host invasion leading to incompatible attachments or tubercle necrosis such as that observed in the sunflower resistant line LR1 (Labrousse, 2001).

Single major resistance genes permit an efficient resistance to diseases. However, genetic resistance based on major dominant genes shows weak sustainability. Breeding for sustainable resistance needs to combine QTLs and major genes (Lindhout, 2002; Palloix et al., 2009; Brun et al., 2010). Additionally, significant gains can be made through breeding approaches informed by increasing understanding of the physiology of the parasitic plant association (Pérez-Vich et al., 2013). Accordingly, the objective of this study was to determine the genetic architecture of quantitative resistance in a RIL population combined with a candidate gene approach based both on race F field phenotyping and race F and G laboratory screening for resistance mechanisms.

## Materials and Methods

### Sunflower Lines and *Orobanche cumana* Races

A population of 101 recombinant inbred lines (RILs) (F_8_) was obtained by single-seed descent from a cross between two parental lines HA89 and LR1. LR1 is an inbred line derived from an interspecific cross involving the wild species *Helianthus debilis* (ecotype 215 in the INRA collection) (Labrousse, 2001). The sunflower line 2603 was used as a susceptible control in each experiment (Labrousse, 2001). All sunflower lines are part of the French genetic resources collection maintained by INRA (crb.tournesol@toulouse.inra.fr). The same population was previously described by Labrousse et al. (2004) for the resistance to *O. cumana* race E. A large variability of response was observed in the population for their capacity to induce the germination of *O. cumana* seeds, to control the development of tubercles and to induce tubercle necrosis. Using *O. cumana* race E, the authors found that LR1sunflower line disables the vascular connection leading to necrosis of 100% established tubercles (Labrousse, 2001). HA89 was found to be more susceptible to *O. cumana* race E than LR1, inducing necrosis only in 60% of the established tubercles but allowing further development of the remaining parasites (Labrousse et al., 2004).

The RIL population was characterized for plant height in Toulouse (France) in an *O. cumana*-free experimental field. The plants were sown in 4 m long rows of 10 plants. Plant height was recorded by measuring the main stem height in five individual plants (in the center of the row) at flowering stage during 2015 growing season.

*Orobanche cumana* seeds, race F, were collected in Marchena (Province of Sevilla, Spain) in 2012. During field experiments in 2014 and 2015, the *O. cumana* population parasitized sunflower line NR5 (carrying the *Or5* gene conferring resistance to race E) but not the P-96 line (conferring resistance to *O. cumana* race F), indicating that this population corresponded to race F (data not shown). *O. cumana* seeds race G were collected in 2000 in an experimental field located at Çeşmekolu (Kirklareli Province, Turkey). This *O. cumana* population was confirmed as race G because it parasitized sunflower lines K-96, P-96 and R-96 (carrying resistance to race F) (Fernández-Martínez et al., 2004).

### Rhizotron Experiments: Evaluation of Resistance to Race F Early Post-vascular Development

*Orobanche cumana* seeds were surface-sterilized with 2.6% NaClO for 5 min and were rinsed thoroughly with sterile water. Race F of *O. cumana* seeds were spread on moistened sterile glass fiber filter paper (GF/A, Whatman) and incubated for 7 days in the dark at 22°C to allow *O. cumana* seed conditioning, a process used in broomrape experiments to break seed dormancy and promote seed sensitivity to molecules inducing germination secreted by host roots (Lechat et al., 2012). The sunflower seeds were first germinated in moistened filter paper for 3 days and then transferred to sand for four additional days. Subsequently, the rhizotron system was used to enable the interaction between 7-days conditioned *O. cumana* seeds and the roots of 7-days old sunflower plants. Briefly, a rhizotron is made of two PVC glasses (12 cm × 12 cm) confining the sunflower roots and the broomrape seeds on a glass fiber filter. Sterilized blocks of rockwool (Grodan, ROCKWOOL) of 1cm of thickness kept the glass fiber filter wet where the root system was deposited. Rhizotrons were placed in a growth chamber under a 16 h photoperiod and a constant temperature of 22°C during day and night periods. Plants were watered daily with half strength Long Ashton Nutrient Solution (LANS, Hewitt, 1966). A balanced incomplete-block design was performed to study the attachment stage. Seventeen blocks of 30 different RILs, permitting the phenotyping of each RIL five times, and the two parental lines HA89 and LR1 were designed using the R package DBI. After 2 weeks of growth, the numbers of compatible attachments (CA) and incompatible attachments (IA) were counted under a stereomicroscope (S6D, Leica). Attachments (stage 2 in **Figure 1**) were considered as compatible when the radicle of *O. cumana* was observed penetrating the host root, characterized by a slight swelling of the *O. cumana* radicle. Attachments were considered as incompatible when the radicle of *O. cumana* had initiated host root penetration but were stopped by host defenses mechanisms. The *O. cumana* radicle was not connected to the vascular system of the host leading to the death of the parasite. In addition, darkening of host cells at the penetration point was observed. The rate of IA was calculated as the percentage of IA out the total of attached *O. cumana* radicles (IA and CA).

### Pot Experiments: Evaluation of Resistance to Race F and G at Young Tubercle Development

The evaluation of each RIL for resistance to *O. cumana* races F and G (stage 3 in **Figure 1**) was performed according to Louarn et al. (2012). Sunflower seeds were first germinated in moistened filter paper for 3 days. The substrate (charred clay for race F and a mixture of sand and vermiculite for race G) was inoculated with 60 mg of *O. cumana* seeds per liter of substrate and was moistened with water allowing the *Orobanche* seeds to undergo conditioning during 7 days. Then, 3 days pre-germinated sunflower seeds were sown in the pot (volume of 70 ml) and kept in a growth chamber under a 16 h photoperiod and a constant temperature of 22°C during day and night periods. Plants were watered daily with LANS (~10 ml/plant). After 5 weeks of growth, the level of infection by *O. cumana* was determined for each RIL by counting the number of healthy and necrotic tubercles on 5 (race F) or 9 (race G) sunflower plants from 5 or 3 independent experiments, respectively. The rate of necrotic tubercle was calculated as the percentage of necrotic tubercle out of the total number of tubercles (healthy and necrotic). The susceptible line 2603 and the race F resistant line P-96 were used as controls.

### Field Evaluation of *O. cumana* Race F Emergence

The RILs were evaluated together with the parental lines for *O. cumana* race F resistance under artificial inoculation in field conditions in the spring and summer of 2014 and 2015 (stage 4 in **Figure 1**). Sunflower seeds were germinated during 2 days in moistened filter paper and subsequently transferred to small pots (7 cm × 7 cm × 7 cm) containing a mixture of sand and peat (1:1, v:v). Previously, soil (~180 g) was carefully mixed with 50 mg of *O. cumana* seeds to obtain a homogeneously infested substrate. The plants were watered by hand as needed and kept in a growth chamber for 15–20 days (time necessary for transplanting all plants to the field) at 25°C/20°C (day/night) with a 14 h photoperiod for incubation. They were then transplanted to a field plot at the experimental farm of the Institute for Sustainable Agriculture (CSIC, Córdoba, Southern Spain) in which only race F experiments have been conducted since 1999. The assays were transplanted between the 31st of March and the 2nd of April 2014 and between the 25th of March and the 27th of March 2015. The evaluation consisted of three replicates of 15 plants by row for each experiment. The plants were set 33 cm apart in 5 m row with 1 m separation between rows. The race F susceptible lines 2603 and NR5, and the resistant line P-96 were used as controls. The number of emerged broomrapes per sunflower plant was counted at the time of sunflower maturity.

### Genotyping

Genomic DNA for the 101 RILs and the two parental lines was extracted using the Kit DNeasy Plant Mini Kit (Qiagen©). The DNA concentration was adjusted to 10 ng.μl^-1^ in water.

The hybridization experiments were performed by the Gentyane platform (Plateforme Gentyane, UMR INRA/UBP 1095 Génétique Diversité et Ecophysiologie des Céréales, 5 chemin de Beaulieu – 63039 Clermont-Ferrand, France) on a GeneTitan^®^ (Affymetrix) according to the manufacturer’s instructions. The AXIOM array was built using a set of 586 985 SNPs in the frame of the SUNRISE project^1^. Genotypic data were obtained with the software Axiom Analysis Suite^2^.

The genotypic data were filtered, and SNPs were selected according to the following criteria: (a) the three replicates of the two parental lines were homozygous and consistent, (b) HA89 and LR1 were polymorphic, (c) the allelic frequency in the population was between 40 and 60%, (d) the missing data in the 101 RILs did not exceed 5%, and (e) the number of the heterozygous data in the whole population did not exceed 5%. We obtained a set of 21 201 SNPs for the genetic mapping. The genetic map was obtained using CarthaGène software (de Givry et al., 2005). After merging the markers (“mrkmerges” function), the LGs were obtained with the “group 0.2 3” function. The genetic distance between markers was calculated using the “lkh 1 -1” function and the genetic maps were obtained with the “bestprintd” function. The resulting genetic map, containing 951 markers obtained from 21 201 heterozygous SNPs, is shown in Supplementary Figure S1. The sequences of the markers mapped are included in Supplementary File S1.

### QTL Mapping

Quantitative trait loci detection was performed with MCQTL (Jourjon et al., 2005) with a threshold corresponding to a Type I error rate of 1% at the genome-wide level, as determined after 3 000 replications of the resampling process for each trait. Biomercator (version 4.2) was used for the visualization of the different QTLs (Arcade et al., 2004).

### Statistical Analysis

The relationship between the rate of IA, the rate of necrotic tubercles, the number of healthy tubercles and the number of broomrape shoot emergences in field in 2014 and 2015 were studied by a PCA analysis. PCA was performed with the package FactoMineR using R software (version 3.1.2) under the Rcmdr environment (version 2.1-7). The Pearson correlation test was performed to determine the R coefficient between the different traits.

### *In Silico* Mapping of Candidate Genes

The candidate gene sequences were obtained from Letousey et al. (2007), Radwan et al. (2008), and Li and Timko (2009). These sequences were used to search for the putative orthologous cDNAs in a sunflower transcriptomic database by BLAST^3^. The sunflower sequences were then used for *in silico* mapping by BLAST alignment to a genetic map obtained from the international consortium of sunflower genome sequencing and composed of 454 contigs^4^. The BLAST results were selected according to the following criteria: (i) identity between the query and the subject sequences greater than 95% and (ii) length of the alignment greater than 400 nt. The colinearity between the two genetic maps was determined by a BLAST alignment of the AXIOM markers linked to the QTLs on the 454 contig database.

## Results

### Phenotypic Evaluation of the RIL Population

#### Evaluation of the Resistance to Race F at the Stage 2 and 3 of Broomrape Development

**FIGURE 2:**
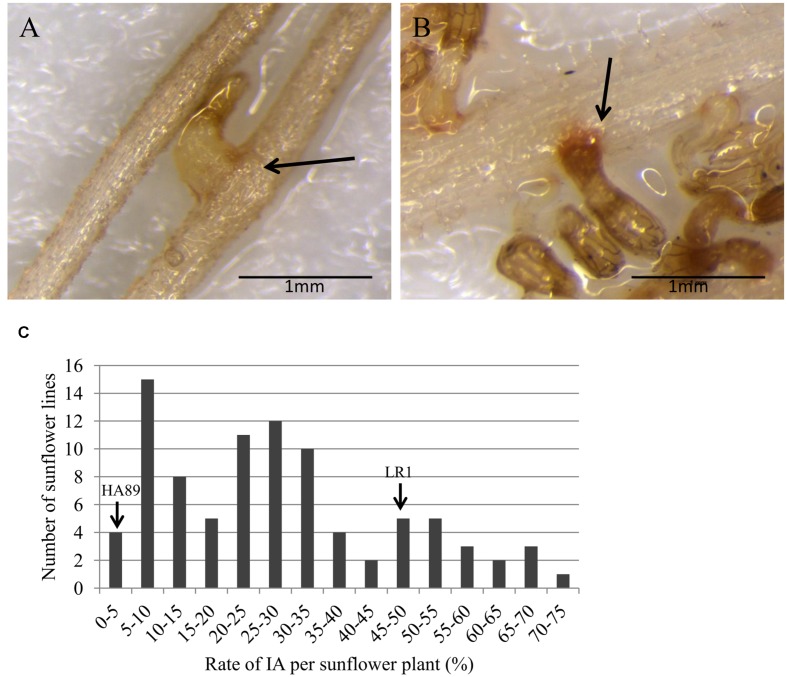
**Description of the resistance mechanism inhibiting *O. cumana* development at stage 2 in the RIL population HA89xLR1.** The photos show **(A)** compatible attachment and **(B)** incompatible attachment between *O. cumana* and sunflower roots. The arrows indicate the attachment area between the radicle of *O. cumana* race F and the sunflower roots. **(C)** Distribution of the rate of IA counted 2 weeks after infection by *O. cumana* race F. The data are calculated from at least 3 replicates for each sunflower line. Replicates with less than 5 attachments (IA + CA) were discarded. The two parental lines HA89 and LR1 showed 3.5 and 46.5% of IA, respectively.

The first physical interaction between sunflower and broomrape occurs underground after seed germination when *O. cumana* fixes to the root system of the host (stage 2, **Figure 1**). This stage was evaluated in rhizotrons, and two different phenotypes were observed. When a sunflower line was susceptible to *O. cumana* race F at this stage, a CA allowed the parasite to attach and connect with the vascular system of the host root (**Figure 2A**). When a sunflower line was resistant, IA was observed. The IA was characterized by a browning of the interaction zone between the parasite and the sunflower root (**Figure 2B**). The two parental lines showed a significant difference for rate of IA at stage 2 (**Figure 2C**). Indeed, minimal IA was observed in the parental line HA89 (3.51%) compared to LR1, which produced a high rate of IA (46.51%). In the segregating population, RILs showed a continuous quantitative resistance profile from susceptible (IA ≤ 10%) to resistant (IA ≥ 50%). Thus, 14 lines showed a high rate of IA (IA ≥ 50%), and a low rate of IA (IA ≤ 10%) was observed for 19 lines (**Figure 2C**).

**FIGURE 3:**
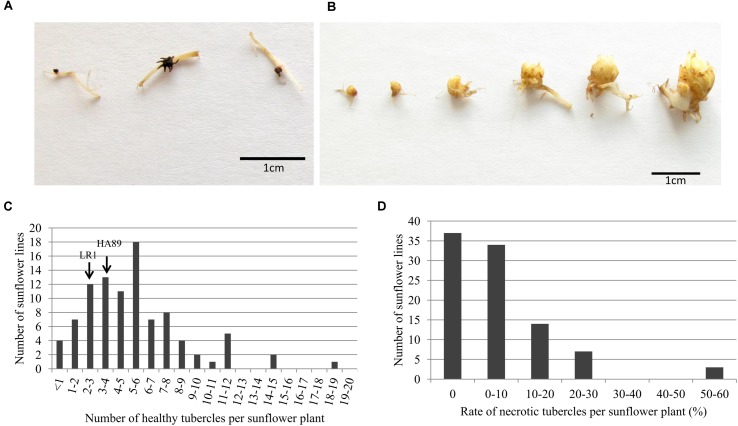
**Evaluation of the resistance level to *O. cumana* race F in the RILs population HA89xLR1 at stage 3.** Photos illustrate the different developmental stages observed for either **(A)** necrotic tubercle or **(B)** healthy tubercle after 5 weeks post infection. **(C)** Distribution of the number of healthy tubercle in the RILs population HA89xLR1 5 weeks after infection. The two parental lines HA89 and LR1 show 3.4 and 2.4 healthy tubercle per plant, respectively **(D)** Distribution of the rate of necrotic tubercle in the RILs population HA89xLR1 5 weeks after infection. No necrotic tubercles were observed for the two parental lines. Value represents the number of RILs for each range of the number of healthy tubercle or rate of necrotic tubercle per sunflower plant. Five independent experiments were performed with one single plant per RIL in each experiment.

Following successful attachment and vascular connection during stage 2, *O. cumana* develops a storage organ called tubercle (stage 3, **Figure 1**). The RIL population was phenotyped in pots for stage 3 development of *O. cumana* race F, by counting the number of healthy and necrotic tubercles attached to each sunflower line (**Figures 3A,B**). The necrosis of the tubercles was characterized by browning and death of the parasite (**Figure 3A**). In contrast, the healthy tubercles remained yellow and became larger, allowing the next stages of development of *O. cumana* (**Figure 3B**). Although the size of the tubercles was not systematically measured, the healthy tubercles observed after 5 weeks were approximately 1 cm in diameter, the bigger tubercles showing a differentiated bud (**Figure 3A**).

The number of healthy tubercles observed in the two parental lines HA89 and LR1 was similarly low: 3.4 and 2.4 tubercles/plant on average, respectively. Despite the low number of *O. cumana* tubercles observed in the two parental lines, the segregating population allowed a wider range of tubercle development (from 0 to 18 tubercles/plant, **Figure 3C**). Some lines exhibited a high resistance level, with less than 1 healthy tubercle in average, similar to the level of resistance in the resistant line P-96. Highly susceptible RILs were also found with a susceptible reaction similar or higher to sunflower line 2603 (12.85 tubercles/plant on average) (**Figure 3C**).

Besides the absolute number of healthy *O. cumana* tubercles per plant, the rate of necrotic tubercles out of the total tubercles developed was also calculated as a possible discriminating mechanism of resistance (**Figure 3D**). Negligible values of necrosis of tubercle were observed in *O. cumana* attached to either parental lines HA89 and LR1 or in the susceptible control line 2603. As mentioned above, *O. cumana* was unable to develop beyond stage 2 in the resistant control P-96 and in consequence no tubercles of any kind (healthy or necrotic) were observed. Most of the RILs induced negligible or low level of necrotic tubercles (<30%), except for 3 RILs that exhibited an average of necrosis higher than 50% (**Figure 3D**).

#### Evaluation of the Resistance to Race G at Stage 3 of Broomrape Development

Race G of *O. cumana* is found only in countries around the Black Sea. The population was not evaluated in field, for precautionary quarantine reasons; therefore the resistance level at stage 3 of tubercle development was measured in a confined growth chamber. For race G, the number of healthy tubercles developing on the susceptible line 2603 was 10.88 tubercles per plant on average and was 7.22 and 8.44 healthy tubercles/plant on average, for HA89 and LR1 respectively. However, the number of *O. cumana* tubercles on the roots of parental lines in race G was more than twice that in race F. The level of infestation was also higher in the RILs population when challenged to race G. Despite no fully resistant RIL (no tubercle development) was observed, a wider range of responses was observed in the RIL population when compared with the parental lines (from 1.14 to 16.55 tubercles/plant, Supplementary Figure S2A). Necrosis of tubercle was not observed except in 8 RILs with an average of tubercle necrosis higher than 20%. (Supplementary Figure S2B). Interestingly, two of these RILs also induced significant necrosis in tubercles when challenged with *O. cumana* race F. There was no correlation for number of healthy tubercles in the sunflower population between the screenings made with race F and race G (Supplementary Figure S2C).

#### Resistance to Race F in the Field

**FIGURE 4:**
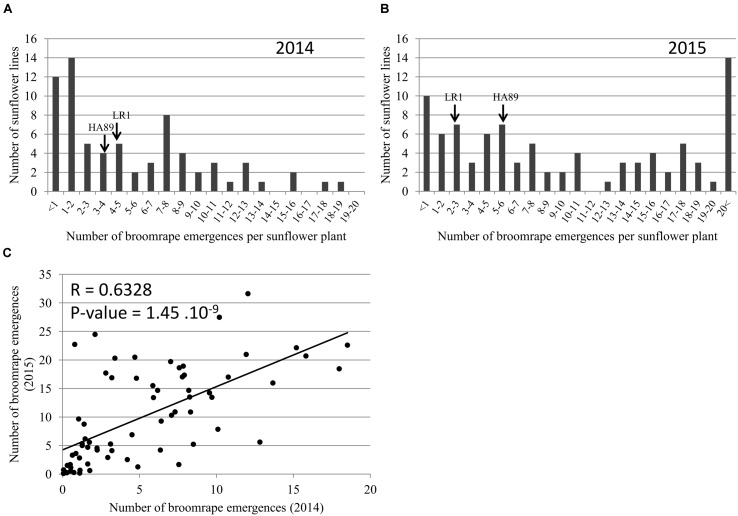
**Characterization of the resistance level in field at the stage 4 on the RILs population HA89xLR1.** Distribution of the number of *O. cumana* emerged shoots per sunflower plant in 2014 **(A)** and in 2015 **(B)**. The data represent the mean of three replicates of 15 sunflower plants in field (Cordoba, Spain). **(C)** Relationship between the number of *O. cumana* emerged shoots in 2014 and 2015.

Phenotyping of the RIL population in the field was performed in 2014 and 2015. In both years, the two parental lines showed a similar resistance profile to *O. cumana* race F (**Figure 4**). A large number of resistant lines were observed in both years (12 and 10 RILs showing less than 1 broomrape emergence per sunflower plant in 2014 and 2015 respectively). In 2015 (**Figure 4B**), the overall intensity of the attack was higher than in 2014 (**Figure 4A**), with a consistent two times (1.94) increase *O. cumana* emergence average per sunflower plant across the entire RIL population allowing to establish a high correlation between experimental years (**Figure 4C**).

Plant size can be related with intensity of *Orobanche* emergence in the field masking genetic resistance. Vigorous hosts can increase the chances of parasitic encounter through bigger root systems or, once *Orobanche* is attached, they can provide better nutritive supply, therefore increasing the rate of parasitic emergence. In order to discover possible associations between sunflower vigor and the levels of *O. cumana* emergence observed in Cordoba, the plant height of the RIL population was characterized in *O. cumana*-free field conditions and its variability (Supplementary Figure S3) was mapped to one QTL on LG 5 (Supplementary Figure S5). The variability observed for sunflower plant height was not significantly related with the level of *O. cumana* emergence in sunflower (Supplementary Figure S4).

#### Correlation between the Resistance Mechanisms at Stage 2, 3, and 4 of Broomrape Development

**FIGURE 5:**
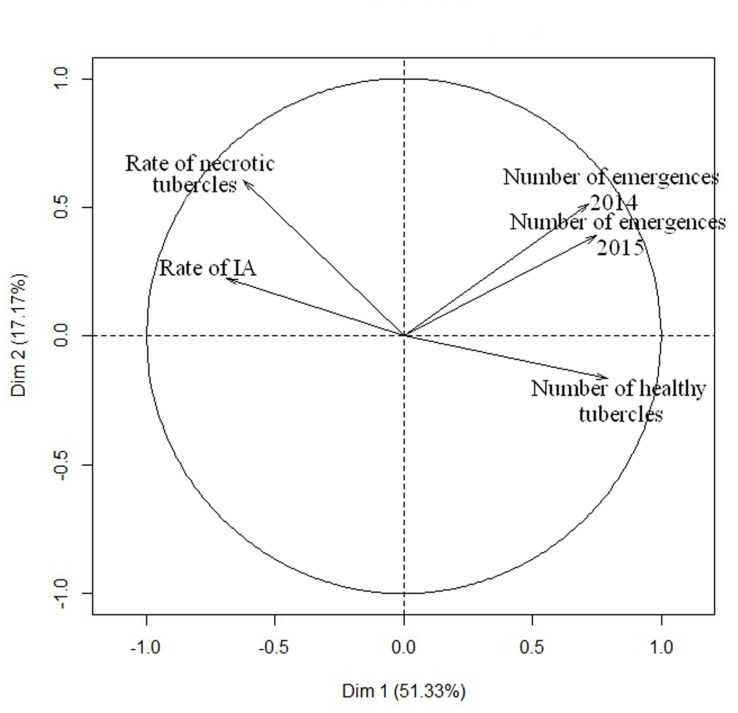
**Relationship between the different sunflower resistance traits to *O. cumana* race F.** PCA was performed with all phenotypic data from the 101 RILs. The two first axis of the PCA explain 68.5% of the variability.

PCA analysis was performed to determine the relationship between the different traits measured for race F of *O. cumana* (**Figure 5**). The first axis of the PCA explained more than 50% of the variability, distinguishing between healthy tubercle development in pots and shoot emergence in field on the basis of the rate of IA and the necrosis rate. This PCA analysis also highlights the correlation between the field observations in 2014 and 2015, as shown in **Figure 4C**, as well as the correlation between susceptibility in the field and the number of healthy tubercles. Interestingly, the rate of IA and the necrosis of the tubercles were negatively correlated to the number of healthy broomrape tubercles and the number of *O. cumana* field emergences. When resistance data to race G was added, no correlation was observed in the RIL population for number of healthy tubercles (data not shown). This result is consistent with the data from Supplementary Figure S1C.

**FIGURE 6:**
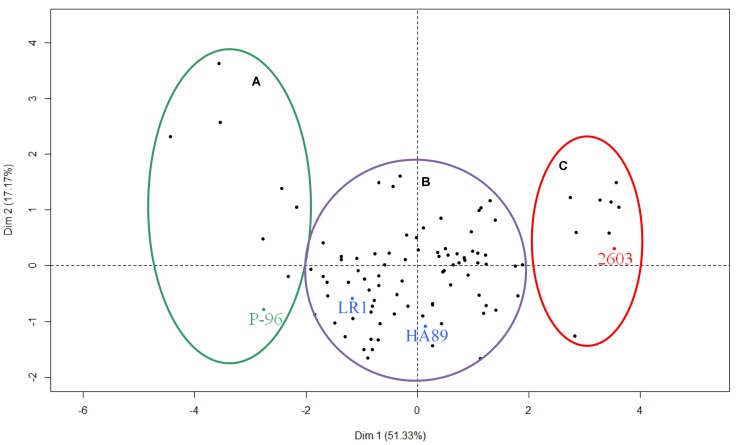
**Phenotypic diversity within the complete RIL population for *O. cumana* race F.** Three groups were found according to the first dimension of the PCA. They correspond to resistant lines **(A)**, susceptible lines **(C)** and partially resistant lines **(B)**. The four control lines HA89, LR1, P-96 and 2603 have been added as additional samples to be shown on the figure but they have not been taken into account in the PCA analysis.

Finally, the different RILs were distributed according to the different axes of the PCA (**Figure 6**). The parental lines HA89 and LR1 and the control lines P-96 and 2603 were added to the figure as supplementary individuals. The population was divided into three distinct groups. The two smaller groups were composed of 7 and 8 RILs and were grouped with the resistant line P-96 and the susceptible line 2603, respectively. The majority of the RILs (86) were grouped together with the two parental lines HA89 and LR1, exhibiting a partial resistance profile to race F of *O. cumana*.

### QTL Mapping

**FIGURE 7:**
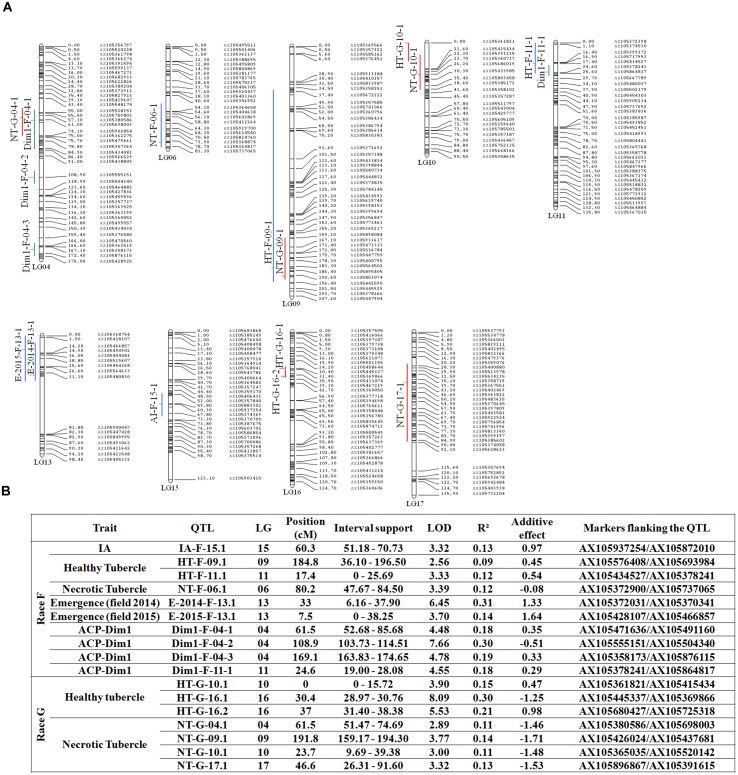
**Quantitative trait loci (QTL) mapping of the different quantitative resistance traits to *O. cumana* race F and G. (A)** The genetic map used to detect QTLs covered the 17LGs (951 genetic bins and 1795.8 cM lengths). QTLs are indicated at the left of each Linkage Group. **(B)** Summary of the different QTL found for the resistance to *O. cumana* race F and G. QTL name nomenclature is: trait-broomrape race-linkage group-QTL number on the linkage group. Additive effect, (-) be equivalent to a decrease of the value due to LR1 alleles. The traits used for QTL mapping were: IA, ratio of incompatible attachments (IA); Emergence, the number of emerged shoot broomrape per sunflower plant under field conditions in 2014 and 2015; Healthy Tubercle, the number of healthy tubercles; Necrotic Tubercle, ratio of necrotic tubercles; ACP-Dim1, the coordinates of the first axis from the PCA analysis. QTL detection was performed with MCQTL (Jourjon et al., 2005) with a threshold corresponding to a Type I error rate of 1%.

The 101 RILs were genotyped using a high-throughput genotyping tool. The genotypic data were used to obtain a genetic map of 1795.8 cM for the 17 LGs of the sunflower genome. Twenty one thousand two hundred and one markers were mapped and located on 951 different genetic bins, with a mean distance between bins of 1.9 cM. Combined with the phenotypic data, the genotypic data were used for mapping the QTLs. A total of 17 QTLs were found to control resistance to *O. cumana* race F and G (**Figure 7**). These QTLs were localized on 9 LGs. None of the QTLs controlling the different resistant traits for race F were mapped in the same region, and only one QTL controlling the resistance to race F and G colocalized on LG 9.

From the 13 QTLs, we identified two QTLs with a strong effect. The first, on LG16, controlled the healthy tubercles of race G of *O. cumana*. The second controlled the number of emergences in the field and was mapped on LG13. This QTL was identified in both years 2014 and 2015 and was the only one controlling resistance in the field.

The coordinate of the first axis of the PCA was used to perform QTL analysis. Four different QTLs were identified. Two of these QTLs localized with QTLs on LG04 and LG11, controlling the number of healthy tubercles for *O. cumana* race F and the rate of necrotic tubercles for *O. cumana* race G, respectively. The other QTLs were also found on LG04 (**Figure 7**).

Analysis of the additive effect showed that the genetic resistance to race F of *O. cumana* at 2 QTLs is coming from LR1 and at 4 QTLs from HA89. The QTLs controlling the number of emergences, E-2014-F-13.1 and E-2015-F-13.1, explain between 14 and 31% of the variability and the resistant alleles are carried by the parental line HA89 (**Figure 7B**). IA is induced by a locus from the parental line LR1 (**Figure 7B**). For the control of race G, the two most important QTLs identified HT-G-16.1 and HT-G-16.2, were found on the same LG and are very close to each other. These two QTLs have 2 distinct parental origins, HT-G-16.1 is from LR1 and HT-G-16.2 is from HA89 (**Figure 7B**).

### Candidate Gene Mapping

We attempted a candidate gene approach, based on previous functional results on the plant–parasite interaction. Nucleotide-binding site leucine-rich repeat (NBS-LRR) proteins play an important role in plant resistance to pathogens (McHale et al., 2006). Radwan et al. (2008) have identified several NBS-LRR homologs in the sunflower. Based on genetic map positions, only one QTL, “IA-F-15.1” on the LG15, colocalized with one NBS-LRR gene identified by Radwan et al. (2008). This NBS-LRR gene was coded by EF559379.1 cDNA.

Li and Timko (2009) were the first to identify gene-for-gene resistance to parasitic plants, and they have identified RSG3-301 and its predicted coiled-coil-nucleotide-binding-site-leucine-rich repeat (CC-NBS-LRR). BlastX analysis of this protein was performed on the sunflower genome to identify its homolog, and the 20 best hits were mapped on the sunflower genome. Interestingly, only three of these homologs colocalized with QTLs identified to control the resistance to *O. cumana*. These homologs colocalized with QTLs controlling broomrape field emergence (on the LG13, HaT13l034464), controlling the capacity to induce IA (on the LG15, HaT13l008311) and controlling the induction of necrotic tubercle on LG17 (HaT13l008327).

Letousey et al. (2007) have identified HaGSL1 to be induced during incompatible interactions between *O. cumana* and the sunflower root of LR1. HaGSL1 was mapped on the LG09 of the sunflower genome, but it was not localized under the QTLs found on this LG.

## Discussion

### QTL Mapping

In this study, few phenotypic differences were observed between the two parental lines HA89 and LR1 for races F and G, as previously observed by Labrousse et al. (2004) for race E. Interestingly, the IA rate observed for *O. cumana* race F, was significantly different between HA89 (3.51%) and LR1 (46.51%). This difference was not observed at stage 3 of *O. cumana* development. It would be interesting to evaluate the two parental lines in a kinetic of *O. cumana* development between 2 to 5 weeks in order to determine the importance of this resistance mechanism. The RILs population was not suitable to map major genes because the two parental lines do not exhibit a clear discriminating resistance profiles at stage 3 and at stage 4 of *O. cumana* development. The parental lines could also be monomorphic for loci controlling some traits that will not allow the detection of these QTLs in our study. However, some recombinant lines obtained from the cross between HA89 and LR1 exhibited a higher level of resistance or susceptibility than the two parental lines, which indicates complex multigenic control of the resistance that can be increased by particular allele combinations for several loci. Velasco et al. (2012) showed that the resistance carried by *H. debilis subsp tardiflorus* was monogenic; indicating that the use of some *H. debilis* accessions in breeding programs could be an additional way to improve resistance to *O. cumana*.

The expression of resistance is complex because it arises from a combination of several resistance mechanisms acting at different steps of broomrape development. We mapped QTLs controlling some of these steps. Even though some traits were correlated (**Figure 5**), we never observed QTLs that colocalized in the same genetic region (**Figure 7**). We identified only one QTL explaining more than 30% of the phenotypic variability in the entire population. Almost all QTLs explained between 9 and 30% of the phenotypic variation of the traits. This result suggests that many minor quantitative loci are involved in the expression of the traits and cannot be detected in our genetic design. The first report identifying QTLs for broomrape resistance in sunflower was provided by Pérez-Vich et al. (2004), who mapped 7 QTLs controlling the resistance to *O. cumana* race F and found that each of them had a contribution (R^2^) varying from 11 to 38%, similarly to those observed in this study. However, the early stages of the interaction (**Figure 1**) were not taken into account in this previous study. We identified a stable and strong QTL on LG13 for resistance in the field, and our results suggest that the final expression of the resistance in the field is not correlated with one specific mechanism but is due to a combination of resistance mechanisms acting at the early stages of broomrape development.

### Race-Specific Quantitative Resistance

The number of healthy tubercles at stage 3 was the trait best correlated with the number of emergences in the field for race F of *O. cumana* (**Figure 5**). The resistance to race G of *O. cumana* was only measured in controlled conditions at stage 3. Compared to race F, twice more tubercles were observed in the RIL population using *O. cumana* race G. These experiments were performed in same controlled conditions (nutrition, light, temperature and hygrometry). Then, the differences found between the 2 races cannot be explained by environmental effects but were due to the higher virulence of race G. No correlation was observed between the number of broomrape tubercles found for race F and G in the RIL population (Supplementary Figure S2C). As expected, the QTL analysis showed that only two QTLs for the resistance of both races colocalized on LG09 (**Figure 7**). The race-specific resistance to broomrape has previously been reported for quantitative loci (Pérez-Vich et al., 2004) and for resistance control by major genes (Vranceanu et al., 1980). The genetic control of the race-specific resistance in the pathosystem *H. annuus*/*O. cumana* is similar to the one found for downy mildew in the *H. annuus*/*P. halstedii* pathosystem (Vear et al., 2007). It would be interesting to further explore the genetic control of the race-specific resistance by dynamic phenotyping of the interaction from the germination of the broomrape seeds to the final steps of parasite development. Such experiments could identify the important steps involved in the interaction for the different races.

### New Phenotyping Tools Need to be Developed

In our study, the induction of iIAs during the early stages of development played an important role in the resistance mechanisms. However, the attachment is a small and difficult structure to observe. New phenotyping tools are necessary for a better characterization of the interaction between the sunflower roots and *O. cumana*. Furthermore, increasing the depth (qualitative) and the throughput (quantitative) of phenotyping would enrich the quality of the genetic analysis. Such phenotyping methods have successfully been used to describe the development of the roots. These rapid and efficient tools have been one of the major challenges of the decade. Several recent reports have helped to improve the characterization of the root architecture (Iyer-Pascuzzi et al., 2010; Clark et al., 2013; Slovak et al., 2014). Despite the difficulty in observing the attachment, the IA shows a browning at the point of attachment between the host and the parasite. These browning contrasts with the sunflower root could be used to detect IA in high definition pictures. Furthermore, the development of high throughput phenotyping tools will allow for the kinetic analysis of the infection, which could detect new resistance mechanisms.

### Effect of the Environment and Sunflower Plant Height on Resistance

The same field in Cordoba (Spain) was used to perform two experiments in 2014 and 2015. We observed a correlation between data obtained for the whole population during both years (**Figure 4C**; Supplementary Figure S4). However, the number of *O. cumana* emergences in 2015 was approximately twice in average the number of emergences in 2014. Temperature has been shown to have an effect on *O. cumana* development. Sukno et al. (2001) and Eizenberg (2003) have found that a moderate increase of the temperature had a positive effect on the intensity of sunflower infection by broomrape. The increase of the infection level between both years could be due to the increased temperature in Cordoba in 2015 compared to 2014. We obtained the climatic data for both years^5^ between March and June. In March 2015, an increase of the mean temperature of 1°C was observed compared to 2014. After transplanting, the higher maximal temperature observed during the first week in 2015 (24.5°C) than in 2014 (17.9°C) could improve the attachment of the parasite to the host root and could explain the differences in the number of emergences between both years. In April, the mean temperature was similar, but the mean temperatures in May and June 2015 were approximately 2°C higher than in 2014. The effect of the temperature on the level of infestation did not affect the genetic control of the resistance. A stable QTL was mapped on LG13 with data from 2014 and 2015 (**Figure 7**). Besides temperature, other environmental factors could influence *O. cumana* development across seasons. For the field experiments, we used an artificial inoculation in controlled conditions during the first 20 days of the sunflower life. New studies should be conducted to take into account the soil management, rain, nutritive input, weeds and soil microflora composition, to provide a complete understanding of the dynamics of the parasitic interaction and to identify the key environmental factors affecting the development of *O. cumana* and thus indirectly the level of resistance in the sunflower.

It has been observed that the host plant vigor was correlated with *Orobanche* shoot emergences in several pathosystems (Fernández-Aparicio et al., 2009b, 2012a; Fondevilla et al., 2010). The same RIL population was evaluated in 2015 in Toulouse (France) for plant height (as estimate of sunflower vigor). Plant height in the RIL population showed a normal distribution (Supplementary Figure S3). **N**o **c**orrelation between plant height and the number of broomrape shoot emergences in field season 2014 and 2015 was observed (Supplementary Figure S4). We mapped a QTL controlling plant height to LG05, where no resistance QTLs was mapped (Supplementary Figure S5). These clues indicate that the resistance to *O. cumana* race F and plant height are not linked in our RIL population HA89xLR1.

### Candidate Genes Located in the QTL Regions

For breeding programs, the stable QTL found on LG13, which controls the number of emergences in the field is the one that could be most rapidly used. Pérez-Vich et al. (2004) have mapped a QTL controlling resistance to race F in the same genetic region using a different mapping population (P-21 × P-96). In our conditions, this QTL explains 15–30% of the overall phenotypic variability, which is similar to the effect of the QTL reported by Pérez-Vich et al. (2004). Furthermore, we identified a cDNA (HaT13l034464), showing homology with a gene coding for a CC-NBS-LRR protein described by Li and Timko (2009) that were located in the interval support of this QTL. NBS-LRR proteins play important roles in gene-for-gene resistance (McHale et al., 2006).

Two NBS-LRR genes (GenBank accession number EF559379.1 and HaT13l008311) were identified in the interval support of a QTL mapped to LG15. One of these genes encodes for a NBS-LRR protein (HaT13l008311) showing homology to RSG3-301 that controls the incompatible interaction between *Striga gesnerioides* and cowpea (*Vigna unguiculata*) (Li and Timko, 2009). These authors found that RSG3-301 induces an early resistance mechanism and acts on the attachment zone between the roots of *Vigna unguiculata* and *S. gesnerioides*. In our study, a similar phenotype (IA) was observed and evaluated for all RILs of the population (**Figure 2**), and one QTL was found to control this resistance mechanism on LG15. The attachment of the broomrape radicle to the sunflower root is an essential step for the parasite to establish the interaction to redirect sunflower assimilates for its growth. However, we show that the sunflower has a resistance mechanism to block this interaction. In some lines, there is a rapidly incompatible interaction mechanism. This response is characterized by browning at the point of attachment between the host and the parasite. A similar incompatible phenotype of the *Orobanche* penetration process has been described in several crop species including *Vicia* spp. response to *O. aegyptiaca* (Goldwasser et al., 2000), and the response of rice (Gurney et al., 2006) or *Sorghum* (Mohamed et al., 2003) to *Striga* spp. Further cytological or biochemical experiments would be needed to investigate the nature of the incompatible phenotype observed in sunflower resistant RILs. Several defense mechanisms could underlay the incompatible phenotype including hypersensitive response (Morel and Dangl, 1997), a rapid and strong resistance reaction usually mediated by specific recognition of pathogen-derived effectors by the cognate resistance protein in the host during a gene-for-gene interaction. To date, the *Orobanche*-encoded molecular cues that determine the resistant phenotype in the host are unknown. Identification of candidate genes by gene expression comparison between compatible and incompatible interactions could provide a better understanding of the interaction mechanism.

With a full sequence of the sunflower genome, we could identify other candidate genes. For all of the candidate genes that we have identified, more experiments are needed, and it would be interesting to test their functional roles in resistance by reverse genetics using EMS mutants (Sabetta et al., 2011; Kumar et al., 2013) or to measure their expression level in different genotypes or during the different steps of the interaction.

## Conclusion

For the first time, the early stages of the *O. cumana*/*H. annuus* pathosystem were used to map QTLs. No pleiotropic QTLs were found and, these QTLs controlled specific developmental stages. To complete the overall panel of possible resistance mechanisms, it is important to characterize the induction of *O. cumana* seed germination by sunflower root exudates and the induction of the haustorium at the genetic level. It was found that the low capacity to induce broomrape seed germination by the host was a good way to control broomrape in field (Jamil et al., 2011; Fernández-Aparicio et al., 2012b). However, Labrousse et al. (2004) have not found a correlation between the capacity to induce broomrape seed germination and the level of infection in the sunflower.

The emergence of new virulent races has frequently been observed, and new race-specific resistance loci need to be identified. Even though the introgression of major resistance genes is an easy and quick solution for breeding, more sustainable resistance has to be developed. In other pathosystems, the additive effect of minor QTLs has improved resistance to broomrape (Fondevilla et al., 2010; Gutiérrez et al., 2013). Breeding methods that integrate QTLs are a good way to improve the sustainability of sunflower resistance to broomrape. Our results show that several quantitative resistance loci can be used. However, the diversity of broomrape populations needs to be detailed, and the emergence of new races and new geographical infected areas must be monitored.

No resistance genes have yet been cloned, and the molecular mechanism underlying the resistance to *O. cumana* is poorly described. One of the main future goals will be to better understand the interaction between the sunflower and *O. cumana*.

## Author Contributions

M-CB and NP contributed to the production of the data. LV and JL contributed to the production and to the analysis of the data. BP-V, PV, and SM contributed to the analysis of the data and to the coordination of the project.

## Conflict of Interest Statement

The authors declare that the research was conducted in the absence of any commercial or financial relationships that could be construed as a potential conflict of interest.

## Footnotes

1 http://www.sunrise-project.fr/en/

2 http://www.affymetrix.com

3 https://www.heliagene.org/HaT13l/

4 http://www.sunflowergenome.org/

5 http://power.larc.nasa.gov
