# Supplementary material for: Sunflower Resistance to Broomrape (Orobanche cumana) Is Controlled by Specific QTLs for Different Parasitism Stages
Source: Front Plant Sci. 2016 May 10;7:590. doi: 10.3389/fpls.2016.00590 (PMC4861731; doi:10.3389/fpls.2016.00590)
Supplement: Supplementary file 1 [file Presentation_1.PPTX]

## Slide 1
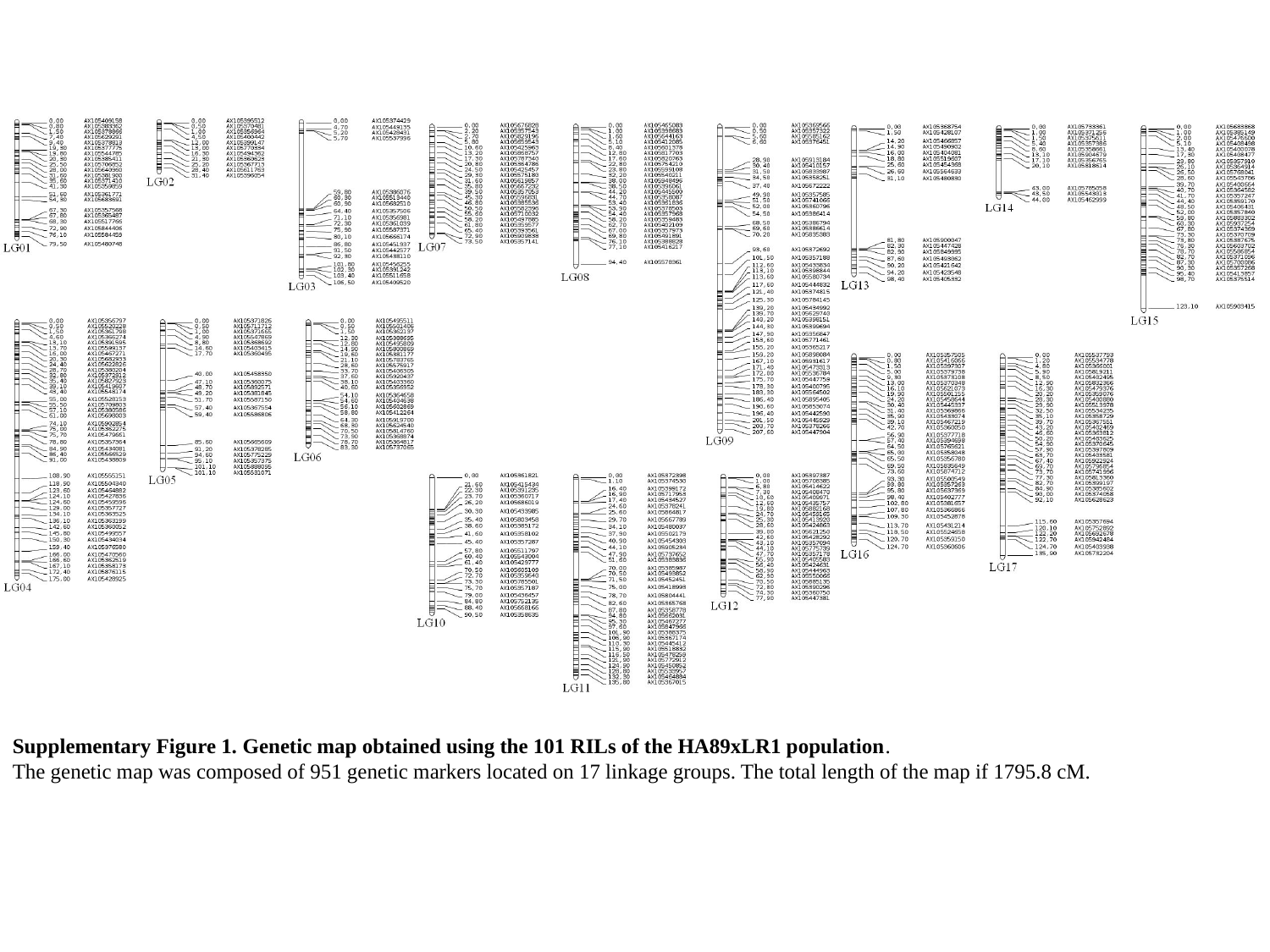

Supplementary Figure 1. Genetic map obtained using the 101 RILs of the HA89xLR1 population.
The genetic map was composed of 951 genetic markers located on 17 linkage groups. The total length of the map if 1795.8 cM.

## Slide 2
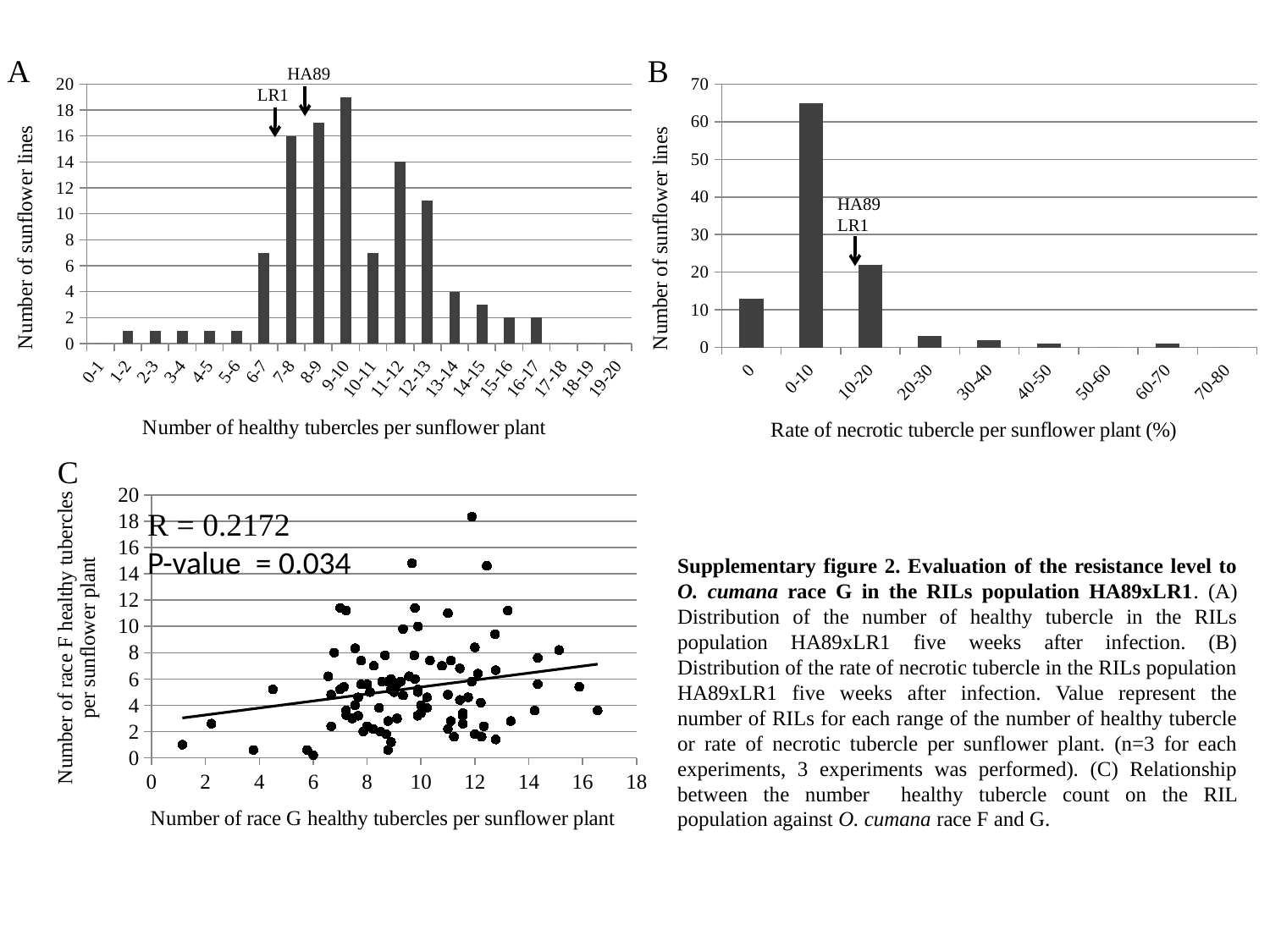

A
B
HA89
### Chart
| Category | |
|---|---|
| 0-1 | 0.0 |
| 1-2 | 1.0 |
| 2-3 | 1.0 |
| 3-4 | 1.0 |
| 4-5 | 1.0 |
| 5-6 | 1.0 |
| 6-7 | 7.0 |
| 7-8 | 16.0 |
| 8-9 | 17.0 |
| 9-10 | 19.0 |
| 10-11 | 7.0 |
| 11-12 | 14.0 |
| 12-13 | 11.0 |
| 13-14 | 4.0 |
| 14-15 | 3.0 |
| 15-16 | 2.0 |
| 16-17 | 2.0 |
| 17-18 | 0.0 |
| 18-19 | 0.0 |
| 19-20 | 0.0 |
### Chart
| Category | |
|---|---|
| 0 | 13.0 |
| 0-10 | 65.0 |
| 10-20 | 22.0 |
| 20-30 | 3.0 |
| 30-40 | 2.0 |
| 40-50 | 1.0 |
| 50-60 | 0.0 |
| 60-70 | 1.0 |
| 70-80 | 0.0 |LR1
HA89
LR1
C
### Chart
| Category | |
|---|---|R = 0.2172
P-value = 0.034
Supplementary figure 2. Evaluation of the resistance level to O. cumana race G in the RILs population HA89xLR1. (A) Distribution of the number of healthy tubercle in the RILs population HA89xLR1 five weeks after infection. (B) Distribution of the rate of necrotic tubercle in the RILs population HA89xLR1 five weeks after infection. Value represent the number of RILs for each range of the number of healthy tubercle or rate of necrotic tubercle per sunflower plant. (n=3 for each experiments, 3 experiments was performed). (C) Relationship between the number healthy tubercle count on the RIL population against O. cumana race F and G.

## Slide 3
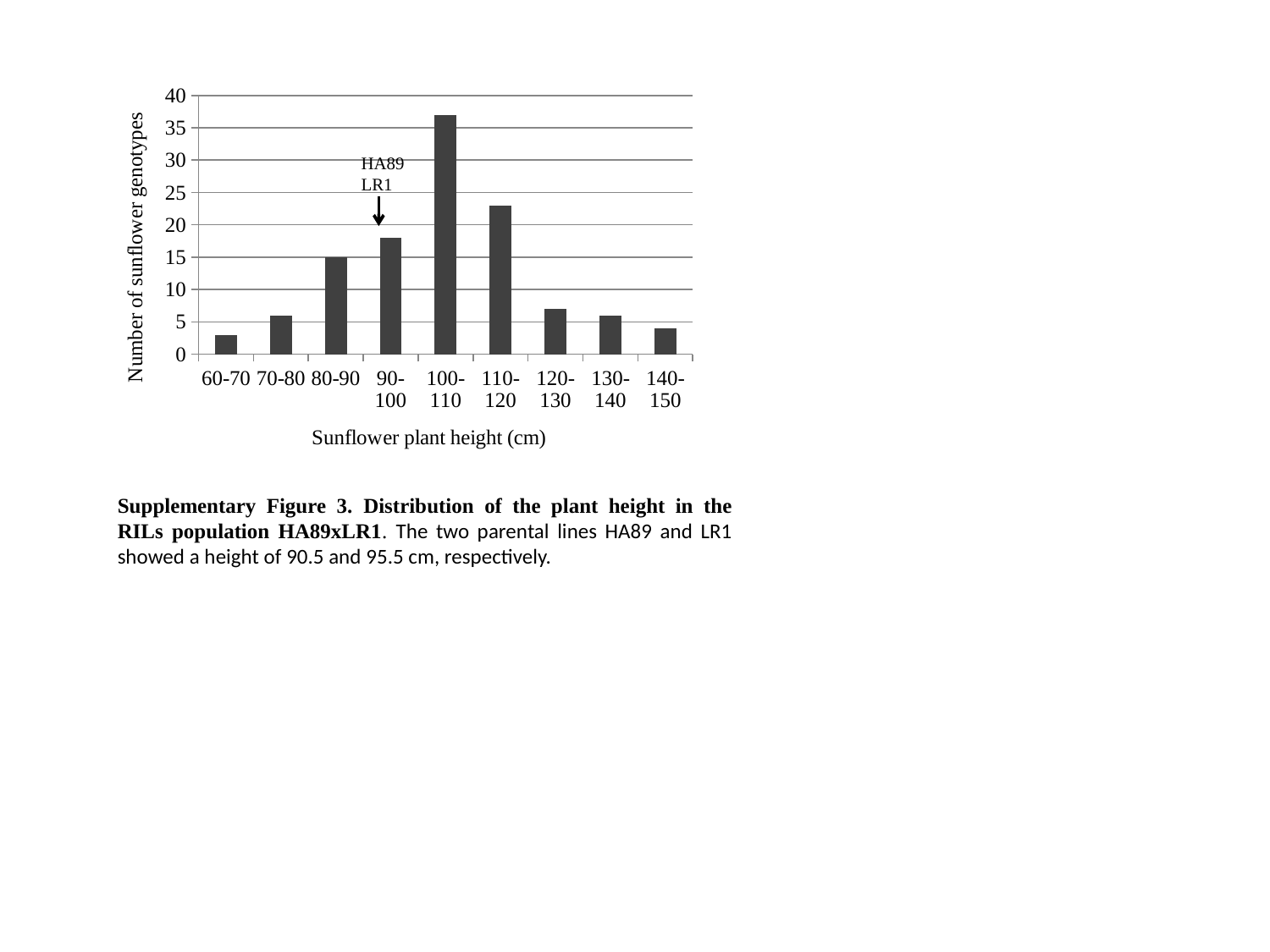

### Chart
| Category | |
|---|---|
| 60-70 | 3.0 |
| 70-80 | 6.0 |
| 80-90 | 15.0 |
| 90-100 | 18.0 |
| 100-110 | 37.0 |
| 110-120 | 23.0 |
| 120-130 | 7.0 |
| 130-140 | 6.0 |
| 140-150 | 4.0 |HA89
LR1
Supplementary Figure 3. Distribution of the plant height in the RILs population HA89xLR1. The two parental lines HA89 and LR1 showed a height of 90.5 and 95.5 cm, respectively.

## Slide 4
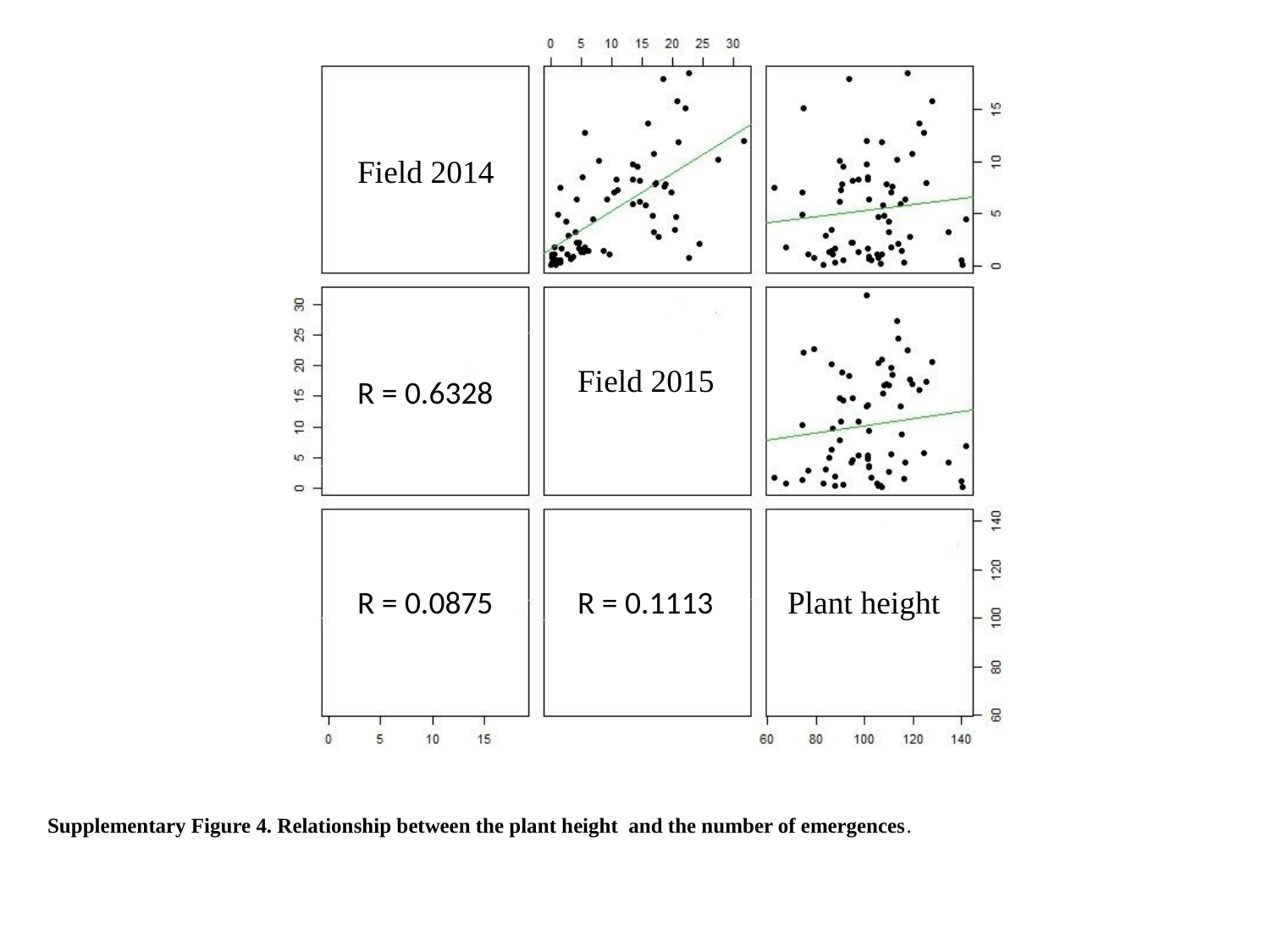

Field 2014
Field 2015
R = 0.6328
R = 0.0875
R = 0.1113
Plant height
Supplementary Figure 4. Relationship between the plant height and the number of emergences.

## Slide 5
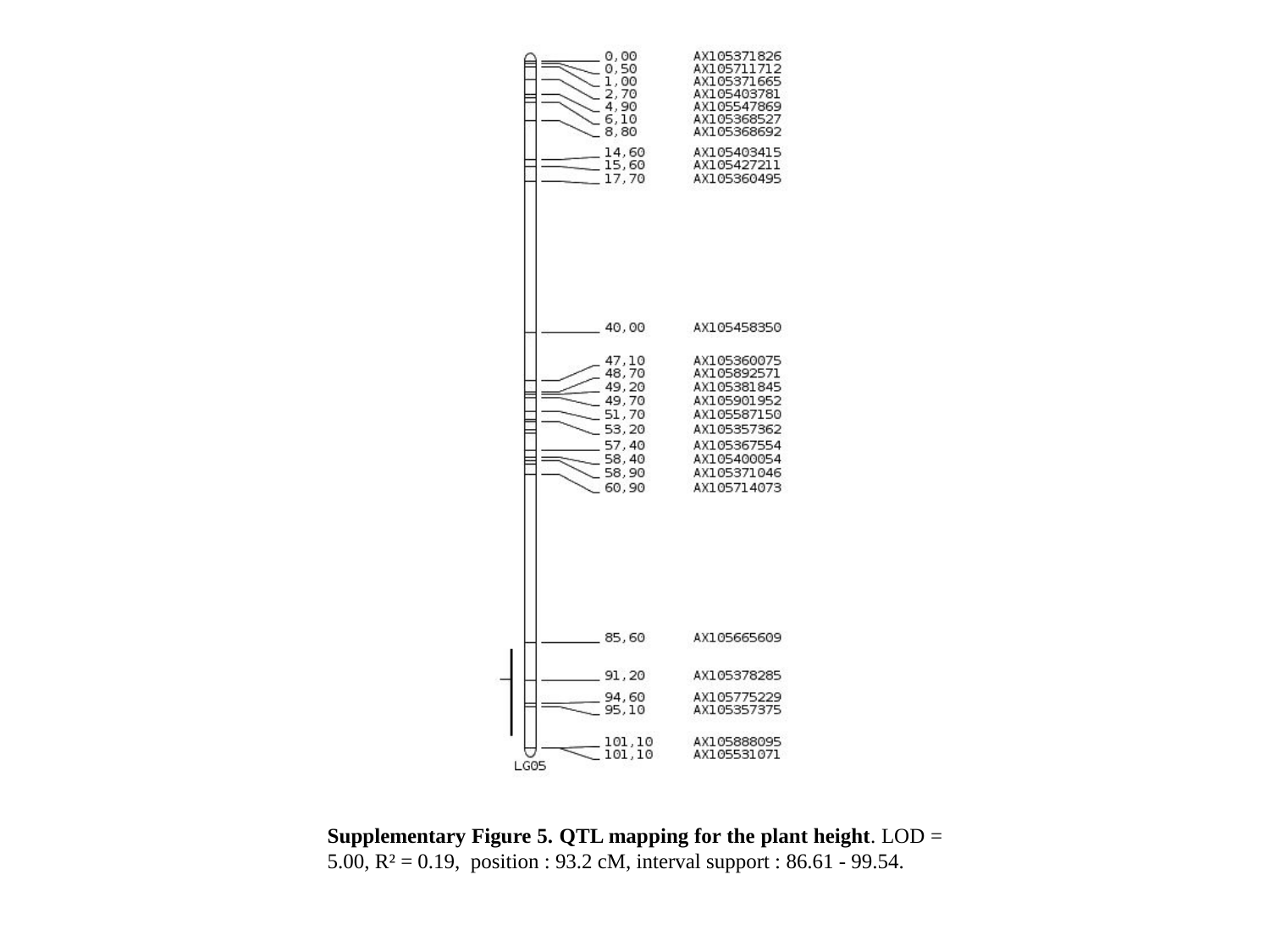

Supplementary Figure 5. QTL mapping for the plant height. LOD = 5.00, R² = 0.19, position : 93.2 cM, interval support : 86.61 - 99.54.
